# Exploring the extent of digital food and beverage related content associated with a family-friendly event: a case study

**DOI:** 10.1186/s12889-021-10716-w

**Published:** 2021-03-30

**Authors:** Ashley Amson, Lauren Remedios, Adena Pinto, Monique Potvin Kent

**Affiliations:** School of Epidemiology and Public Health, University of Ottawa, Ottawa, ON K1N 7K4 Canada

**Keywords:** Digital marketing, Food, Beverages, Family-friendly event, Social media, Youth, Case study, Social norms

## Abstract

**Background:**

Exposure to unhealthy food and beverage content is a contributing factor to the obesity epidemic. Youth are susceptible to unhealthy digital food marketing including content shared by their peers, which can be as influential as commercial marketing. Current Canadian regulations do not consider the threat digital food marketing poses to health. No research to date has examined the prevalence of food related posts on social media surrounding family-friendly events. The aim of this study was to explore the frequency of food related content (including food marketing) and the marketing techniques employed in social media posts related to a family-friendly event in Canada.

**Methods:**

In this case study, a content analysis of social media posts related to a family-friendly event on Facebook, Twitter, and Instagram was conducted between January to February 2019. Each post containing food related content was identified and categorized by source and food category using a coding manual. Marketing techniques found in each food related post were also assessed.

**Results:**

A total of 732 food and beverage related posts were assessed. These posts were most commonly promoted through Instagram (*n* = 561, 76.6%) with significantly more individual users (61.5%; *p* < 0.05) generating food and beverage related content (*n* = 198, 27%) than other post sources. The top most featured food category was fast food (*n* = 328, 44.8%) followed by dine-in restaurants (*n* = 126, 17.2%). The most frequently observed marketing techniques included predominantly featuring a child in the post (*n* = 124, 16.9%; *p* < 0.0001), followed by products intended for children (*n* = 118, 16.1%; *p* < 0.05), and the presence of family (*n* = 57, 7.8%; *p* < 0.0001).

**Conclusions:**

The present study highlights the proliferation of unhealthy food and beverage content by individuals at a family-friendly event as well as the presence of food marketing. Due to the unfettered advertising found in digital spaces, and that they are largely unregulated, it is important for future policies looking to combat childhood obesity to consider incorporating social media into their regulations to safeguard family-friendly events. General awareness on the implications of peer to peer sharing of unhealthy food and beverage posts should also be considered.

**Supplementary Information:**

The online version contains supplementary material available at 10.1186/s12889-021-10716-w.

## Background

Childhood obesity is a growing public health concern. As of 2017, roughly 30% of Canadian youth, aged 5 to 17, were considered overweight or obese [1]. Childhood obesity poses a significant public health risk, as it is often a precursor to negative health outcomes during adulthood [2]. Children and youth experiencing obesity are at a higher risk for several comorbidities including cardiovascular disease, type II diabetes, asthma, and depression [2]. Obesity rates are correlated with an increased consumption of unhealthy foods and beverages. The overconsumption of these products is partly attributable to aggressive marketing tactics and the sharing of food related content across multiple media platforms, such as television and digital media, and settings, such as schools and recreation centres [3]. Systematic reviews demonstrate that exposure to unhealthy food and beverage related content is a contributing factor to the childhood obesity epidemic [4]. Given the negative impact that digital marketing and exposure has on children and youth, it is alarming that social media spaces are largely unregulated in Canada.

Children and youth are heavily exposed to unhealthy food and beverage marketing in digital media [5]. Digital marketing is a ubiquitous form of marketing that capitalizes on the use of digital technologies, such as the internet or social media applications (e.g. Facebook, YouTube, Instagram, and Twitter), to deliver commercial messages that target specific audiences on their smartphones, tablets, and computers [6]. It is characterized by the interactivity and customization of messages, as well as the ability to disseminate product information using targeted methods and volumes beyond the capacity of traditional media [6, 7]. Potvin Kent et al. [5] found that almost three-quarters of Canadian children and youth aged 7–16 were exposed to digital food and beverage marketing via their favourite social media applications. On average, children were exposed to food and beverage marketing on these applications an estimated 30 times per week, while adolescents were exposed to an average of 189 unhealthy food and beverage advertisements per week [5]. Such results are concerning given that emerging research has identified digital marketing as a determinant of child and youth eating habits and dietary patterns [4]. Digital marketers have developed numerous influential marketing techniques to target audiences, such as personalized marketing messages and encouraging peer-to-peer marketing [6, 8].

The sharing of food and beverage related content amongst peers has demonstrated to have repercussions on youths’ consumption of unhealthy food and beverage products. Peer, as defined by Niu, is a “group of members who know each other, share mutual knowledge and life experience, and serve as a comparison or reference to each other [9]. Murphy and colleagues [10] conducted research indicating that adolescents perceived peers who shared unhealthy food and beverages in their social media feeds more positively than those who did not. Youth emulate the marketing they are exposed to, creating and sharing posts on social media that mimics popular food and beverage marketing campaigns [11]. The use of hashtags and prompts to “like”, “share”, and tag others by one’s peers is an additional measure of spreading content across social media platforms quickly and powerfully [12] to a diverse network of people [13]. Ultimately, the proliferation of food and beverage related content that is shared by peers can be likened to youth being recruited as peer marketers. Peer-to-peer marketing is when peers share and promote brands and products either intentionally or not [14, 15]. Exposure from peer-to-peer marketing contributes to the overall marketing environment, as youth prefer to share unhealthy food and beverage related content compared to healthy food or non-food related items [10].

Content that is shared by peers can be impactful, leading to habit forming behaviours.

Woisetschläger et al. [16] draw links between peer groups and purchasing habits, as the importance of social bonds within a group can impact patterns of purchasing and consumption. The food and beverage choices promoted within the digital realm are mostly unhealthy and the cyclical nature of reinforced habits perpetuated by today’s youth leads to the increased likelihood of unhealthy food and beverage consumption amongst peer groups. The influence of peers is more predominant during adolescence as youth shift from being influenced by their parents to being motivated and swayed by their friends [17]. Peer influence, combined with the surreptitious blending of digital marketing and entertainment, hinder youth from disengaging from messages and mechanisms digital marketers employ to control youths food and beverage preferences and consumption [18].

The sharing of unhealthy food and beverage related content from peer-to-peer and the ubiquity of this content across social media applications has been shown to contribute to normative views of unhealthy products [19]. Normative influence is exerted when behaviour is imitated as a result of certain behaviours being viewed as a necessary or normal behaviour in a given context [19]. Posting photos and videos on social media provides a space for individuals to engage in the creation and (re)distribution of personalized content. As other users repeat the normative behaviour and share it across social media, more people are exposed to the marketing driven behaviour. This process is repeated, reinforcing the normative perception [20]. Youth that spend a large portion of their time on social media increase their exposure to these norms and behaviours. Approximately 93% of adolescents spend up to 5 h per day on social media applications [21], with social media usage increasing amongst those in grades 4 to 10 [22]. Increased usage of social media compounded by their developmental stage and concerns of peer acceptance, make adolescents a prime target by food and beverage companies [20]. Understanding these norms and behaviours is important to understanding the effects of digital marketing and for creating targeted policies aimed at children and youth.

Family-friendly events, an event that tailors its activities towards families and children [23], provide an avenue for creating a normative narrative around food and eating, given the numerous marketing opportunities these events present and the age ranges they cater to. Marketing opportunities at these events include outdoor advertising by event-related sponsors, (e.g. signage), the presence of food and beverage vendors, and a social media presence to advertise the event [3]. No research, to our knowledge, has examined the sharing of food and beverage content over social media that is associated with a family-friendly event. Given the increasing popularity of social media as a source of exposure to food and beverage marketing and content to children and youth, the normative producing capabilities of social media, and the observed link between food and beverage marketing and exposure on childhood obesity, it is crucial that research explores the prevalence and context of digital food and beverage exposures in family-friendly events to inform policies regarding obesity prevention. The objectives of this study were to examine the frequency of food and beverage related content (including food and beverage marketing) across three popular social media applications related to a large family-friendly event and to observe any food and beverage related marketing techniques employed in the various social media posts related to this event.

## Methods

This case study examined the extent of digital food and beverage related content (including food and beverage marketing) associated with Winterlude from January to February 2019. Winterlude is an annual winter festival unique to Ottawa, Ontario, Canada, which attracts thousands of visitors each year [24]. It is promoted as a family-friendly event, featuring various ice sculptures, snow playgrounds with ice slides, skating, and sporting events. Winterlude is government run, overseen by the National Capital Commission, a federal government agency. In 2016, the average group size visiting Winterlude was 5 people inclusive of 1–2 children, with most children being under the age of 10 [24].

A content analysis of food and beverage related content associated with Winterlude that was shared on three social media applications—Facebook, Twitter, and Instagram—was conducted. Official Winterlude affiliated accounts (@capitalexperience) on all three applications, and posts linked to #Winterlude and #Winterlude2019 hashtags on Instagram and Twitter were monitored daily for food and beverage related content (including food and beverage marketing). Food and beverage related content was defined as any social media content (i.e. photos, text) that included branded and unbranded food or beverage products or food brands (without actual products). Any posts that met this criteria were recorded and a screenshot of the post and associated caption (if applicable) was saved. A screenshot was saved of any instance of food or beverage related content that appeared in a video, but the entire video was not saved. For Instagram, only stories saved as “highlights” were reviewed. Stories are a feature of Instagram that allows users to share videos or photos in a slideshow format. Stories are automatically deleted after 24 h; however, they can be permanently saved on a user’s profile as “highlights”. All stories that were not saved as highlights were excluded from review. Each social media post containing food and beverage content was reviewed for the number of views, likes, or retweets it received. Facebook video posts only displayed the number of likes. Posts that did not display one or more of these features were coded as “not applicable”. Each post was documented in an Excel spreadsheet and given a unique identification number. Additional features were recorded including: (a) the social media application on which the post was identified; (b) the hashtag associated with the post (e.g., #Winterlude or #Winterlude2019), if applicable; (c) the social media account responsible for the post (e.g., official Winterlude account, business accounts), sponsored individuals (e.g. an individual who is paid to make a post on social media), non-profit groups (i.e., accounts representing a community, neighbourhood), and/or accounts that do not fit into the previous categories (including hospitals, schools, etc., and individual accounts); (d) the date of the post; (e) the associated caption/text of the post; (f) post type (e.g., video, slide show, static picture), mixed post type (e.g. post features a video and a static picture), and other (e.g. GIFs); (g) name of the featured product and/or brand (multiple companies featured in a post were recorded in the same row; if a post featured a food or beverage product without any associated food or beverage company visible in the post, it was coded as “unspecified” under the food or beverage company type; the top five food and beverage companies (food and beverage companies with the greatest frequency of food or beverage content posts) (h) whether the post featured a specific “product” or a “brand” (i.e. logo); and (i) the link to the post.

Each instance of food and beverage related content was categorized by food or beverage company if applicable and categorized using a coding manual adapted from research previously conducted by Potvin Kent et al. [16] Food and beverage categories included: fast food (any establishment where food is served at a counter); dine-in restaurants, sweet baked goods (e.g., cakes, cookies, etc.); candy and chocolate (including gum); energy drinks; soft drinks; other sweetened beverages (e.g., drinks with added sugar, such as hot chocolate or sweetened coffee drinks); coffee or tea; diet soft drinks; 100% fruit juice; alcohol; bread; breakfast cereals; snack foods (e.g., chips, popcorn, etc.); unknown/other beverages; assorted dishes/meals (e.g., pizza, breakfast, salads); vegetables and fruits; water; and other (e.g., food products that do not fit within any of the aforementioned food categories such as meat products, cheese, etc.).

Each post containing food and beverage related content was assessed for presenting commonly used marketing techniques. Common techniques that are frequently used include: posts prominently featuring a child (0–12 years) or an adolescent (13–17 years); the presence of a family; use of promotional characters appealing to children (e.g., spokes characters such as Tony the Tiger or licensed characters like Spiderman); whether the product was intended for children (e.g., candy, fun-shaped foods); the presence of price promotions (e.g. 2 for 1 drinks); free product giveaways (e.g., complimentary drinks, free hot dogs, etc.); encouragement of peer-to-peer marketing (e.g. “tag your friend”); contests; and “celebrity” endorsed posts (e.g., professional athlete, politician, or local news host). The presence of Winterlude sponsorship activity (e.g., signs, branded giveaways, competitions) of official Winterlude sponsors (Tim Hortons, Pepsi Canada, Rideau Carleton Raceway, Ontario Lottery and Gaming Corporation, Enbridge, Nokia, Air Miles, and the Royal Canadian Mint) were also assessed. All coding of social media posts was conducted by one reviewer as all coding decisions were considered objective, with the exception of the age of individuals within the reviewed posts. Youth that looked younger than 12 years old were coded as children while youth appearing to be over 12 years old were coded as adolescents. The researcher conducting the coding is well versed in the digital marketing of food and beverages and is well positioned to objectively assess the food and beverage related content that was associated with Winterlude.

Frequenices of advertisements and marketing techniques were compared by applications, source, sponsors, and category using SPSS v.26.0. Chi square tests or Fisher’s exact tests with the Freeman-Halton extension for contingency tables larger than 2 × 2 were conducted, as appropriate, to assess the differences in the proportion of food and beverage posts by post source. Monte Carlo estimations with 10,000 simulations were used to determine the *p*-value, using SAS v.9.4. Post hoc analysis using pairwise comparisons with Bonferroni corrections was also performed using SPSS. A statistical significance level of α < 0.05 was adopted for all statistical analyses.

## Results

### Characteristics of social media food and beverage marketing exposures by source

A total of 732 posts were identified as containing food and beverage related content. Examples of observed food and beverage related content are available in the supplemental information (see Supplemental Figures 1-3).

As shown in Table 1, the distribution of food and beverage marketing instances by social media application differed significantly (*p* < 0.0001) between post sources, with food and beverage related content most commonly promoted through Instagram (*n* = 561, 76.6% of all food posts) followed by Twitter (*n* = 152, 20.8%), and then Facebook (*n* = 19, 2.6%). Over half of the food and beverage marketing instances were posted by individuals (*n* = 450, 61.5%), followed by business accounts (*n* = 198, 27%). A total of 500 posts (68.7%) were liked 1–50 times, while 190 posts (26.1%) were liked 51–200 times (Table 2). The proportion of likes on social media posts also significantly differed (*p* < 0.0001) by post source.

**Table 1:** Frequency of food and beverage related content linked to Winterlude by social media application

Social media application	Winterlude (***n*** = 37)	Businesses (***n*** = 198)	Sponsored Individuals (***n*** = 7)	Non-profit organizations (***n*** = 40)	Individuals (***n*** = 450)	Total (***n*** = 732)
Facebook	19 (51.4)^a^	0 (0.0)	0 (0.0)	0 (0.0)	0 (0.0)	19 (2.6)
Twitter	6 (16.2)^a,c^	63 (31.8)^a,b^	4 (57.1)^a,b^	22 (55.0)^b^	57 (12.7)^c^	152 (20.8)
Instagram	12 (32.4)^a^	135 (68.2)^b^	3 (42.9)^a,b^	18 (45.0)^a,b^	393 (87.3)^c^	561 (76.6)

Significant differences between social media application and post source were derived by Fisher’s Freeman-Halton test using a Monte Carlo estimation of 10,000 samples to determine the *p* value, < 0.0001(95%CI < 0.0001–0.0005). Letters within rows denote proportions that differ significantly according to post-hoc pairwise comparisons with Bonferroni correction

**Table 2:** Frequency of likes for food and beverage related content linked to Winterlude by source

Number of likes	Winterlude (***n*** = 33)	Businesses (***n*** = 198)	Sponsored Individuals (***n*** = 7)	Non-profit organizations (***n*** = 40)	Individuals (***n*** = 450)	Total (***n*** = 728)
0 likes	4 (12.1)^a^	7 (3.5)^a,b^	0 (0.0)	1 (2.5)^a,b^	4 (0.9)^b^	16 (2.2)
1–50 likes	26 (78.8)^a,b^	111 (56.1)^a^	4 (57.1)^a,b^	33 (82.5)^b^	326 (72.4)^b,c^	500 (68.7)
51–200 likes	3 (9.1)^a^	70 (35.4)^b^	1 (14.3)^a,b^	5 (12.5)^a^	111 (24.7)^a,b^	190 (26.1)
Over 200 likes	0 (0.0)	10 (5.1)^a,b^	2 (28.6)^a^	1 (2.5)^a,b^	9 (2.0)^b^	22 (3.0)

Significant differences between number of likes and post source were dervied by Fisher’s Freeman-Halton test using a Monte Carlo estimation of 10,000 samples to determine the *p* value, < 0.0001(95%CI < 0.0001–0.0005). Letters within rows denote proportions that differ significantly according to post-hoc pairwise comparisons with Bonferroni correction. All Instagram stories (where it is not possible to “like” story content) were excluded from this analysis

Additional information on the number of views and retweets by post source is available in Supplementary Tables 1 and 2.

### Most promoted food and beverage categories by source

The most frequently posted food and beverage category by the Winterlude accounts, sponsored individuals, non-profit groups, and individuals was fast food (Table 3).

**Table 3:** Ranking of food and beverage categories promoted in social media posts linked to Winterlude by source

	Winterlude accounts (***n*** = 37)	Businesses (***n*** = 198)	Sponsored individuals (***n*** = 7)	Non-profit organizations (***n*** = 40)	Individuals (***n*** = 450)	Total (***n*** = 732)
	n (%)	n (%)	n (%)	n (%)	n (%)	n(%)
**1**	Fast food 21 (56.8)	Dine-in restaurants 80 (40.4)	Fast food 4 (57.1)	Fast food 21 (52.5)	Fast food 249 (55.3)	Fast food 328 (44.8)
**2**	Energy drinks 8 (21.6)	Fast food 33 (16.7)	Candy 2 (28.6)	Candy 9 (22.5)	Candy 82 (18.2)	Dine-in restaurants 126 (17.2)
**3**	Candy 2 (5.4)	Sweet baked goods 23 (11.6)	Dine-in restaurants 1 (14.3)	Alcohol 4 (10.0)	Dine-in restaurants 42 (9.3)	Candy 105 (14.3)
**4**	Other 2 (5.4)	Alcohol 20 (10.1)	Meals 1 (14.3)	Dine-in restaurants 3 (7.5)	Other beverage 35 (7.8)	Other beverage 50 (6.8)
**5**	Veg and fruit 2 (5.4)	Other beverage 13 (6.6)		Sweet baked goods 3 (7.5)	Alcohol 25 (5.6)	Alcohol 49 (6.7)

It was possible for more than one food category to be present in a single social media post

Overall, the top five most featured food categories were fast food (*n* = 328, 44.8%), followed by dine-in restaurants (*n* = 126, 17.2%), candy (*n* = 105, 14.3%), other beverages (*n* = 50, 6.8%), and alcohol (*n* = 49, 6.7%). Both fast food and dine-in restaurants were the most featured food categories in business-related posts (*n* = 80, 40.4% and *n* = 33, 16.7%, respectively), while fast food and candy were the most common among individuals’ posts (*n* = 249, 55.3% and *n* = 82, 18.2%, respectively). Less than 2% of total food posts featured any vegetables or fruit (*n* = 5, 0.7%) or fruit juice (*n* = 3, 0.4%).

### Top five promoted food and beverage companies

The top five most promoted food and beverage companies are highlighted in Table 4.

**Table 4:** Top five food and beverage sponsors promoted in social media posts linked to Winterlude by source

Food companies	Winterlude (***n*** = 29)	Businesses (***n*** = 156)	Sponsored individuals (***n*** = 7)	Non-profit organizations (***n*** = 27)	Individuals (***n*** = 297)	Tota (***n*** = 516)	***P***-value
Beavertails	5 (17.2)^a,b^	13 (8.3) ^a^	3 (42.9)^b,c^	17 (63.0)^c^	203 (68.4)^c,d^	241 (46.7)	< 0.0001
Tim Hortons	19 (65.5)^a^	3 (1.9) ^b^	1 (14.3)^a,b,c^	4 (14.8)^c^	35 (11.8)^c,d^	62 (12.0)	< 0.0001
Wasabi Ottawa	0 (0.0)	21 (13.5) ^a^	0 (0.0)	0 (0.0)	0 (0.0)	21 (4.1)	< 0.0001
Red Bull	8 (27.6)^a^	1 (0.6)^b^	0 (0.0)	0 (0.0)	4 (1.3) ^b^	13 (2.5)	< 0.0001
Starbucks Canada	0 (0.0)	4 (2.6) ^a^	0 (0.0)	0 (0.0)	5 (1.7) ^a^	9 (1.7)	0.8458
Other Companies	0 (0.0)	118 (75.6) ^a^	3 (42.9)^a,b^	8 (29.6)^b^	65 (21.9)^b,c^	194 (37.6)	< 0.0001

Differences in post features were compared between account types using the chi square test for Beavertails and the Fisher’s exact test as appropriate for all other companies (including “other companies). Letters within rows denote proportions that differ significantly according to post-hoc pairwise comparisons with Bonferroni correction. It was possible for more than one food company to be present in a single social media post. Any social media posts without any food company identified were excluded from this analysis

Significant differences in the proportion of ads by post source were observed for Beavertails, Tim Hortons, Wasabi Ottawa, Red Bull, and other companies (*p* < 0.0001). Of these, Beavertails was the most commonly advertised food and beverage company (*n* = 241, 46.7%) followed by Tim Horton’s (*n* = 62, 12%). Posts by individuals had a significantly greater proportion of mentions related to Beavertails (*n* = 203, 68.4%) compared to any other food company. Food and beverage related content promoting Beavertails was more likely to be posted by individuals (*n* = 203, 68.4%) than any other social media account type. Red Bull posts accounted for 28% of official Winterlude site posts. Some of the food and beverage companies under “Other Companies” that were also frequently promoted included Three Brothers Shawarma (*n* = 8, 1.6% of all food posts), Bier Markt (*n* = 6, 1.2%), and Absolut Vodka (*n* = 7, 1.4%). Other companies were calculated as the sum of food and beverage related instances from all other food companies that appeared in posts. There were 99 companies deemed as “other” with each company’s posts contributing 1.55% or less of all food and beverage content related posts.

### Presence of marketing techniques in food and beverage related posts

Overall, the most frequently observed marketing techniques included predominantly featuring a child in the post (*n* = 124, 16.9%), followed by products intended for children (*n* = 118, 16.1%), and the presence of family (*n* = 57, 7.8%). Of the products intended for children, maple taffy (*n* = 88, 74.6%) and fun-shaped cookies (*n* = 18, 15.3%) were the most frequently featured products. Almost a quarter of posts by individuals prominently featured a child (*n* = 107, 23.8%), while only 4.2% (*n* = 19) featured an adolescent (Table 5). Similarly, a significantly greater proportion of posts by individuals featured a family (*n* = 52, 11.6%) compared to other post sources. By comparison, other post features such as contests (*n* = 5, 0.7%), celebrity endorsements (*n* = 7, 1.0%), peer-to-peer marketing (*n* = 8, 1.1%), and use of promotional characters (*n* = 10, 1.4%), significantly differed by post source.

**Table 5:** Frequency of marketing features linked to Winterlude by post source

Post feature	Winterlude (***n*** = 37)	Businesses (***n*** = 198)	Sponsored individuals (***n*** = 7)	Non-profit organizations (***n*** = 40)	Individuals (***n*** = 450)	Total (***n*** = 732)	***P***-value
Child prominently featured (% yes)	4 (10.8) ^a,b^	5 (2.5) ^a,^	0 (0.0)	8 (20.0) ^b,c^	107 (23.8) ^c^	124 (16.9)	<.0001*
Adolescent prominently featured (% yes)	2 (5.4) ^a^	0 (0.0)	0 (0.0)	0 (0.0)	19 (4.2) ^a^	21 (2.9)	0.0076**
Presence of family (% yes)	3 (8.1) ^a^	0 (0.0)	0 (0.0)	2 (5.0) ^a^	52 (11.6) ^a^	57 (7.8)	<.0001**
Product intended for children (% yes)	2 (5.4) ^a^	22 (11.1) ^b^	0 (0.0)	10 (25.0) ^b^	84 (18.7) ^b^	118 (16.1)	0.013*
Use of price promotions (% yes)	0 (0.0)	15 (7.6) ^a^	0 (0.0)	2 (5.0) ^a^	0 (0.0)	17 (2.3)	<.0001**
Free product giveaway (% yes)	0 (0.0)	12 (6.1) ^a^	0 (0.0)	2 (5.0) ^a,b^	3 (0.7) ^b^	17 (2.3)	0.0008**
Encouragement of peer-to-peer marketing (% yes)	0 (0.0)	7 (3.5) ^a^	0 (0.0)	1 (2.5) ^a^	0 (0.0)	8 (1.1)	0.0012**
Contest (% yes)	0 (0.0)	4 (2.0) ^a^	0 (0.0)	0 (0.0)	1 (0.2) ^b^	5 (0.7)	0.1616**
Celebrity endorsements (% yes)	0 (0.0)	1 (0.5) ^a^	1 (14.3) ^b^	0 (0.0)	5 (1.1) ^a^	7 (1.0)	0.1286**
Use of promotional characters (% yes)	1 (2.7) ^a^	3 (1.5) ^a^	0 (0.0)	1 (2.5) ^a^	5 (1.1) ^a^	10 (1.4)	0.3973**

Differences in post features were compared between account types using the chi square test or Fisher’s exact test as appropriate. Letters within rows denote proportions that differ significantly according to post-hoc pairwise comparisons with Bonferroni correction. It was possible for more than one post feature to be present in a single social media post

*p-values derived from chi square test

**p-values derived from Fisher’s exact test

Food and beverage posts featuring a family were mostly identified in posts by individuals (*n* = 52, 11.6%) over other post sources. By comparison, other post features such as contests (*n* = 5, 0.7%), celebrity endorsements (*n* = 7, 1.0%), peer-to-peer marketing (*n* = 8, 1.1%), and use of promotional characters (*n* = 10, 1.4%) cumulatively made up only 4.2% of all food and beverage posts.

## Discussion

This study provides a snapshot of the different types of food and beverage related content and food and beverage marketing that was promoted on three social media applications surrounding a family-friendly event. Our results highlight the proclivity of food and beverage posts, the nature of the featured products and companies, and provides insights into the common marketing techniques that were apparent in posts associated with Winterlude. Most importantly, we identified that: 1) individual users generated more food and beverage related content than businesses; 2) the most frequently featured food category was fast food restaurants; and 3) children were frequently featured in food and beverage posts.

### Individual users posted more food and beverage related content than businesses

One of the most significant findings of this study is that posts were primarily generated by individual users, as opposed to companies. Individual user-generated content also had the most engagement, as measured by the proportion of posts that had at least one like. This is indicative of a trend that has become apparent to public health professionals - individual users are becoming an important and effective source of sharing and promoting food and beverage products online [8]. Food and beverage companies, including fast-food chains, often enlist consumers through social media to promote branded posts amongst consumers social networks [6]. Consumers that send branded posts to their peers are participating in a technique known as peer-to-peer marketing. Recent evidence suggests that peer endorsement of unhealthy food and beverage products is equally as powerful as food companies’ paid endorsements at spreading poor dietary norms on social media [13, 25]. Additional research has identified social media influencers as a significant source of food and beverage promotion [25]; however, food and beverage posts by sponsored individuals were reported considerably less in our sample.

Social norms can be created when individuals generate online content that is broadly shared. As users repeatedly demonstrate a behaviour and share it over social media, more people are exposed to that behaviour and repeat the process, reinforcing the normative perception. Posting unhealthy food and beverages related to a family-friendly event can negatively impact an individual’s health and wellbeing [6]. Although this study did not examine youths’ actual exposure to these food and beverage posts, their potential to adversely affect youths’ dietary behaviours remains, as children and youth are chronic users of social media. Further research is required to understand the extent to which norms are created through the perpetual sharing of food and beverage products on social media, especially amongst children and youth.

### The prominence of fast food and candy-related posts

Fast foods were the most frequently featured food category in our study with almost half of food related posts featuring this food category. The prominence of fast food marketing has also been noted in research exploring advertising on television and on children’s preferred websites [26, 27]. Amongst children and youth, fast food consumption is strongly associated with an increased risk of excess weight or obesity [28]. Other evidence has also connected fast food consumption in the development of additional chronic diseases, including diabetes and cardiovascular disease [29].

Candy was also observed to be a prominent food category across all post sources, with the exception of businesses. Recent digital food marketing research conducted in Canada on child and youth preferrred social media applications has also revealed low nutrient foods, such as candy, to be among the most commonly advertised foods by food companies [5]. Longitudinal evidence supporting the association between adverse health outcomes and increased candy consumption during childhood or adolescence is inconsistent [30, 31]. Nevertheless, the consumption of added sugars has been linked to lower overall diet quality in Canadian youth [32], which is considered a significant risk factor for diabetes and cardiovascular disease [33].

The frequency of alcoholic beverage related content that is apparent in social media postings found in our data is noteworthy and concerning given that Winterlude is a family-friendly event. There is a dearth of evidence in Canada on youth exposure to the digital marketing of alcoholic beverages; however, some research has shown that alcohol was a top 10 advertised product on children’s preferred websites in Canada [27]. Such marketing does impact youth. A systematic review of 12 long-term, international studies established a positive correlation between exposure to alcohol marketing and drinking behavior in youth [34]. Further, results from individual studies illustrated a clear link between increased exposure to alcohol marketing and early initiation of drinking, in addition to engagement in binge drinking [34].

This trend of promoting unhealthy foods and beverages through social media was apparent in posts by Winterlude accounts, where the three most frequently promoted food and beverage categories were fast food, energy drinks, and candy. Winterlude accounts are operated by the National Capital Commission, a federal government agency that oversees tourism and urban planning in Canada’s capital (Ottawa) [35]. These results point to an area of concern, whereby individuals or organizations in positions of authority, especially publicly funded organizations, promote low-nutrient foods and beverages to a wide audience, implying that certain products or brands are endorsed by federal organizations. Much like the impact of social media influencers or peer-to-peer marketing, food and beverage marketing through publicly funded accounts could potentially exert negative influence on food behaviours and habits among youth. It is important that venues, especially government run family-friendly events, are cognizant of the products they are promoting. Promotion of nutrient poor products are likley to be shared by those attending, and in the case of youth, these products will be shared with those in their network perpetuating a narrative of eating low quality, nutrient poor food.

### Children being featured frequently in food and beverage posts

The results of our content analysis revealed that the bulk of social media posts that exhibited food and beverages depicted child-related features and themes (e.g. presence of children or products intended for children) and products most consumed by children (maple taffy and fun shaped cookies). This does not come as a surprise, as Winterlude is advertised as a family-friendly event. Notably, posts of child-related features and themes were shared most by individuals, followed by businesses. Even though individuals sharing food and beverage related content is not a direct form of commercial marketing, sharing food and beverage related content on social media can impact those viewing this content similarly. This is concerning as posts featuring children consuming unhealthy products is likley to generate and contribute to societal eating norms. Previous research has also indicated that adolescents often associate branded food images on social media with social contexts and attach social meanings to branded products [11], contributing to the contextualization of food norms amongst older youth.

Research has demonstrated that even when older children and adolescents are able to accurately recognize the corporate promotion of unhealthy products, they are often unable to resist its influence when the content is personalized or relatable [36]. This notion underscores the importance of policy solutions that recognize the potential impact of food and beverage vendors associated with a family-friendly event and the repercussions these chioces can have on the eating behaviours of children and youth when shared through social media platforms. This is perhaps even more relevant when the events are run by government or organizations where their purpose is to create healthy environments for all, especially children and youth.

### Limitations and strengths

To our knowledge, this is the first study to provide an in-depth examination of food and beverage related posts on social media associated with a family-friendly event. Our findings, which are based on a specific, local event in Ottawa, are not representative of food posts associated with other family-friendly events. The percentage of food and beverage posts as a percentage of all social media posts related to Winterlude was not assessed due to the high volume of all Winterlude-related social media posts and the time intensive nature of this type of data collection. The coding of all instances of food and beverage related content may also have been susceptible to reporting and coding errors, as all data collection and coding was completed by one research assistant. This study also did not examine actual exposure to these social media posts; thus, it is not possible to know exactly which age groups were exposed to these posts. Moreover, this research did not evaluate the healthfulness or nutritional value of featured food and beverage products, which could provide a more granular analysis of the type of diet that is being promoted online due to this event. Despite these limitations, this study provides the first examination into the frequency of social media food and beverage related content associated with a family-friendly event.

## Conclusion

The presence of food and beverage posts shared across three social media applications during Winterlude demonstrates that family-friendly events are an opportune space for the sharing of unhealthy food and beverage related content. Current Canadian regulations fail to recognize the threat that nutrient poor food and beverage exposures at family-friendly events may pose to health, particularly that of children and youth. Future policies for combating childhood obesity through digital channels should consider incorporating social media and family-friendly events into their regulations. Further, greater public awareness is needed to highlight the implications of peer to peer sharing of unhealthy food and beverage posts.

## Supplementary Information

**Additional file 1: Table S1.** Frequency of views for food and beverage social media posts linked to Winterlude by source. **Table S2.** Frequency of retweets for food and beverage social media posts linked to Winterlude by source. **Fig. 1.** Example of a food and beverage social media post linked to Winterlude from Facebook. **Fig. 2.** Example of a food and beverage social media post linked to Winterlude from Instagram. **Fig. 3.** Example of a food and beverage social media post linked to Winterlude from Twitter.

## Data Availability

All data generated or analysed during this study are included in this published article and its supplementary information files.
