# Supplementary material for: Exploring the extent of digital food and beverage related content associated with a family-friendly event: a case study
Source: BMC Public Health. 2021 Mar 30;21:621. doi: 10.1186/s12889-021-10716-w (PMC8011127; doi:10.1186/s12889-021-10716-w)

Title: Exploring the extent of digital food and beverage related content associated with a family-friendly event: A case study

Authors: Ashley Amson, Lauren Remedios, Adena Pinto, Monique Potvin Kent

Affiliation:

University of Ottawa

School of Epidemiology and Public Health

Ottawa, ON K1N 7K4

Canada

Email/phone number of corresponding author:

Dr. Monique Potvin Kent: 613-562-5800 ext. 7447 / [mpotvink@uottawa.ca](about:blank)

**Supplemental Tables and Figures**

Table S1 – Frequency of views for food/beverage social media posts linked to Winterlude by source

| **Number of views** | **Winterlude (n=10)** | **Business (n=7)** | **Sponsored Individuals (n=1)** | **Non-profit organizations (n=2)** | **Individuals (n=7)** | **Total (n=27)** |
| --- | --- | --- | --- | --- | --- | --- |
| ≤ 50 views | 0 (0.0) | 1 (14.3) | 0 (0.0) | 0 (0.0) | 0 (0.0) | 1 (3.7) |
| 50 > views | 10 (100.0) | 6 (85.7) | 1 (100.0) | 2 (100.0) | 7 (100.0) | 26 (96.3) |

Table S2 – Frequency of retweets for food/beverage social media posts linked to Winterlude by source

| **Number of retweets** | **Winterlude (n=6)** | **Business (n=63)** | **Sponsored Individuals (n=4)** | **Non-profit organizations (n=22)** | **Individuals (n=57)** | **Total (n=152)** |
| --- | --- | --- | --- | --- | --- | --- |
| ≤ 50 retweets | 6 (100.0) | 63 (100.0) | 4 (100.0) | 21 (95.5) | 57 (100.0) | 151 (99.3) |
| 50 > retweets | 0 (0.0) | 0 (0.0) | 0 (0.0) | 1 (4.5) | 0 (0.0) | 1 (0.7) |

**Figures**

**Fig. 1** Example of food/beverage social media post linked to Winterlude from Facebook


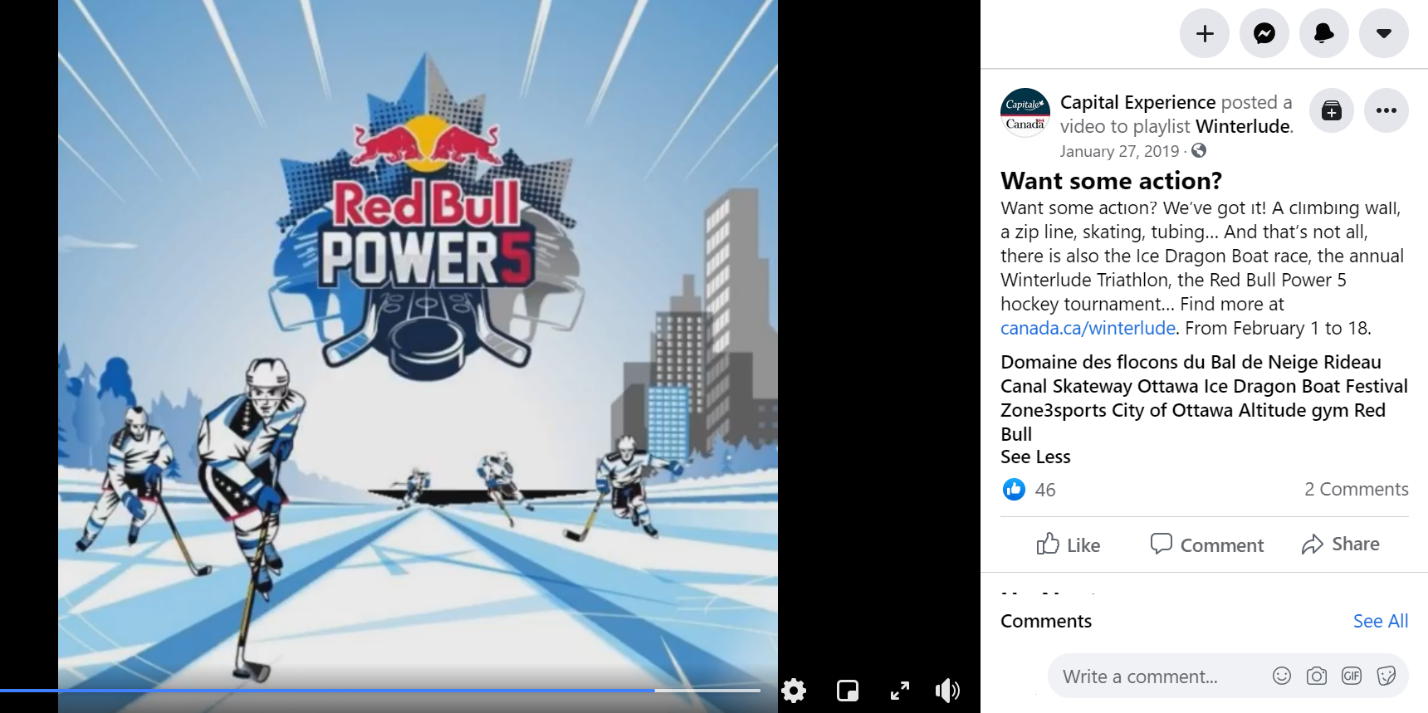


**Fig. 2** Example of food/beverage social media post linked to Winterlude from Instagram


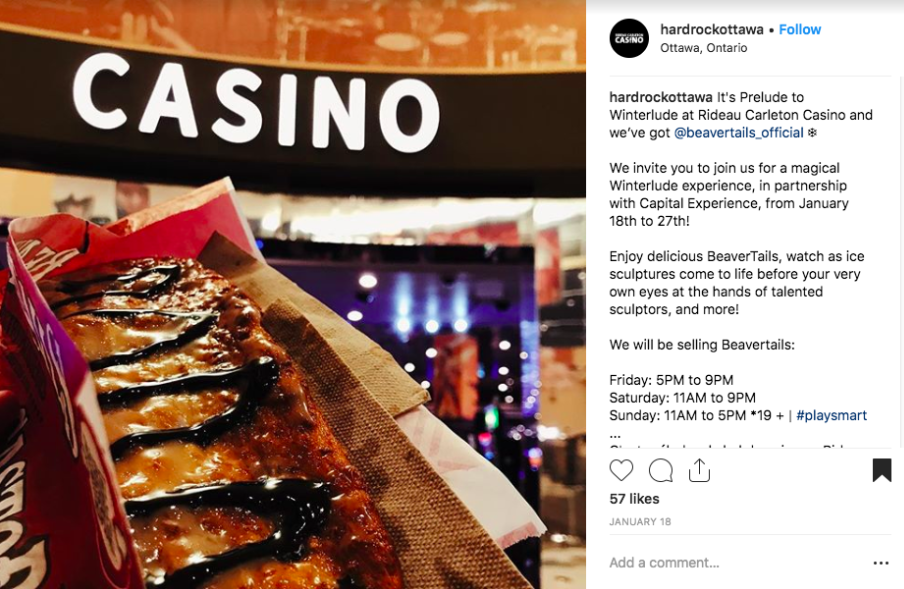


**Fig. 3** Example of food/beverage social media post linked to Winterlude from Twitter


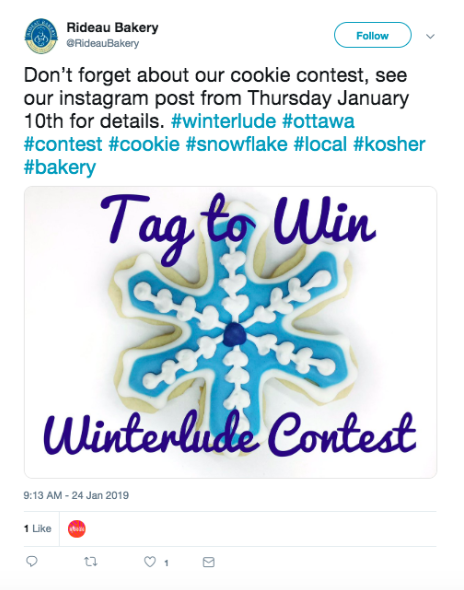

Supplement: Supplementary file 1 — Additional file 1: Table S1. Frequency of views for food and beverage social media posts linked to Winterlude by source. Table S2. Frequency of retweets for food and beverage social media posts linked to Winterlude by source. Fig. 1. Example of a food and beverage social media post linked to Winterlude from Facebook. Fig. 2. Example of a food and beverage social media post linked to Winterlude from Instagram. Fig. 3. Example of a food and beverage social media post linked to Winterlude from Twitter. [file 12889_2021_10716_MOESM1_ESM.docx]
